# Covid-19 vaccination adherence and correlated factors in patients with substance use disorders

**DOI:** 10.1093/eurpub/ckac131.351

**Published:** 2022-10-25

**Authors:** F Mondera, GFM Direnzo, V Cammalleri, FM Forestiero, F Turatto, V Baccolini, C Marzuillo, M Barra, P Villari

**Affiliations:** 1 Department of Public Health and Infectious Diseases, Sapienza University of Rome, Rome, Italy; 2 Fondazione Villa Maraini, Rome, Italy

## Abstract

**Background:**

Analyzing trends and patterns of vaccination during COVID-19 pandemic is important to understand how current policies are working. Evidence regarding the general population is already available, but hard-to-reach populations as migrants or minorities, but also people suffering from a Substance Use Disorder (SUD), have not been fully explored. To understand the extent of vaccine adherence and evaluate associated factors in this population subgroup, we conducted a cross-sectional study in collaboration with Villa Marini Foundation, national agency of the Italian Red Cross for pathological addictions.

**Methods:**

We developed a questionnaire regarding COVID-19 vaccination, adherence to prevention measures, concerns about COVID-19 and questions relating to drug use that we administered to each participant. Only people aged over 18 with a diagnosis of SUD were included in the study. A multivariable logistic regression model was built to identify the predictors associated with anti-SARS-CoV-2 vaccination, estimating adjusted odds ratios (aOR) and 95% confidence intervals (CIs).

**Results:**

We recruited 200 participants between December 2021 and January 2022. Most respondents were male (84.5%), Italian (72%), aged 44 years on average. A total of 40 patients (20%) reported they haven't received any dose of COVID-19 vaccine; the most common motivations were lack of trust in the vaccine and fear of side effects. At multivariable analysis, the use of heroin appears to be negatively associated with vaccination acceptance (aOR=0.31 CI 95%: 0.11-0.81) as well as not being Italian (aOR=0.27 CI 95%: 0.12-0.63).

**Conclusions:**

The vaccination rate in our sample was consistent with the one of the general Italian population in the same period, whereas the reasons behind the lower adherence to vaccination in the heroin group need to be further investigated. It’s also important to reduce possible bureaucratic obstacles that could explain the lower number of vaccinated foreign citizens.

**Key messages:**

• People with Substance Use Disorder are a fragile and often overlooked population that needs to be considered during Public Health intervention and vaccination campaigns.

• The access to vaccination for foreigners should be made more simple and easier to increase accessibility and participation.

